# Psychosocial transformation in women new to fitness: a phenomenological study on body image, self-confidence, and identity construction

**DOI:** 10.3389/fpsyg.2026.1882501

**Published:** 2026-09-16

**Authors:** Mehdi Duyan, Gamze Ok, Ilker Gunel, Mehmet Bayansalduz, Vlad Adrian Geantă

**Affiliations:** 1 Faculty of Sports Sciences, İnönü University, Malatya, Türkiye; 2 Department of Physical Education and Sports, Institute of Health Sciences, İnönü University, Malatya, Türkiye; 3 School of Physical Education and Sports, Osmaniye Korkut Ata University, Osmaniye, Türkiye; 4 Faculty of Sports Sciences, Topkapı University, Istanbul, Türkiye; 5 Faculty of Physical Education and Sport, Aurel Vlaicu University of Arad, Arad, Romania

**Keywords:** body image, fitness, identity construction, phenomenology, qualitative research, self-confidence, women

## Abstract

**Introduction:**

Despite the increase in gym memberships in recent years, women’s participation in physical activity continues to lag behind men’s. Studies that holistically address the experiences of women newly starting fitness are limited in the literature. Aiming to fill this gap, this research seeks to explore in depth the gym experiences of women who have recently started fitness, and the psychosocial transformations they undergo in their body perceptions, self-confidence, and self-narratives throughout this process.

**Method:**

The study was designed within the framework of a phenomenological approach, one of the qualitative research designs. The study group consisted of 16 women who were members of women-only fitness centers in Malatya, Türkiye, and had been exercising regularly for at least two months. Participants’ ages ranged from 23 to 48, with 75% (*n* = 12) having been engaged in fitness for one year or less. Data were collected in written form using a semi-structured interview form; no audio recordings were made due to participants’ privacy concerns. The obtained data were analyzed using Braun and Clarke’s thematic analysis method.

**Results:**

The analysis revealed four main themes: (1) Driving Forces for Initiation, (2) Paradigm Shift in Body and Self-Perception, (3) The Dual Face of the Social Environment, and (4) Transformation Materialized in Daily Life and Identity Construction. The findings indicate that the process of starting fitness holds meaning beyond mere physical activity for the participants; this journey, initiated by medical diagnoses, bodily dissatisfaction, or emotional needs, was associated with profound psychological transformations such as increased self-confidence, reconciliation with the body, and feelings of strength and competence. While the women-only gym served as a safe haven and space for solidarity for the participants, questions from the outside environment such as “why do you exercise?” reveal the persistent influence of social norms.

**Conclusion:**

Ultimately, participants perceived their fitness experiences as contributing to a stronger female identity through autonomy, boundary-setting, and psychological resilience. This research reveals the multidimensional nature of these transformative experiences and emphasizes that physical activity programs should be designed considering these psychosocial dimensions.

## 1 Introduction

Women’s participation in physical activity continues to lag behind men’s, despite the increase in gym memberships in recent years (Cowley and Schneider, 2025; Khairunissya et al., 2025; Rosaini et al., 2026). This situation underscores the need to understand the invisible barriers and social pressures that women face in gym settings. The literature has examined the barriers women encounter in gyms from various perspectives. For instance, Khairunissya et al. (2025), in their systematic review, identified body image pressure, objectification, appearance concerns, and access/cost issues as the biggest barriers to women’s gym participation. Shaheen (2025), in a qualitative study with Palestinian women, demonstrated that gender norms and cultural expectations constitute significant barriers to women’s participation in strength sports. Similarly, women have been reported to frequently feel judged by their appearance and performance in gyms, constantly feeling “inadequate” and having to cope with unwanted comments from men (Cowley and Schneider, 2025). According to Rosaini et al. (2026), women’s participation in strength training is shaped by four main factors: health concerns, performance goals, various barriers (time, motivation, cultural pressures, belief in competence, accessibility issues), facilitators such as social support, and developed strategies. Despite all these adversities, women demonstrate resistance against gender norms and appearance ideals in gyms, and some women can experience empowerment through skill acquisition, breaking gender norms, and exercising in supportive environments (Cowley and Schneider, 2025; Csontos et al., 2026; Rosaini et al., 2026).

When women’s motivations for exercise participation are examined within the framework of objectification theory and self-determination theory, gender roles and body perception emerge as determining factors (Nelson and Jette, 2023; Yuan et al., 2025). According to objectification theory, the perception of women’s bodies as sexual objects in Western culture is associated with women evaluating themselves from a third-person perspective, which may negatively affect their exercise motivations (Frederick et al., 2022; Paulisova and Orosova, 2023; Murofushi et al., 2025). Self-determination theory, on the other hand, suggests that when individuals’ basic psychological needs for autonomy, competence, and relatedness are met, their intrinsic motivation increases, as does the likelihood of continuing exercise (Deci and Ryan, 2002; Zhang et al., 2024; Deininger and Teriba, 2025; Ntoumanis and Moller, 2025).

## 2 Theoretical background

### 2.1 Digital platforms and social media effects

Pressures on women’s body perception extend beyond the physical boundaries of gyms to digital platforms. An experimental study examining the effect of exposure to fitness posts on social media on women’s body esteem found that fitness posts, particularly when containing appearance-oriented content, increase body surveillance and decrease body esteem in women (Yan et al., 2025). Galway et al. (2026) also demonstrated that watching “what I eat in a day” videos on TikTok triggers upward appearance comparisons, especially among young adults, and increases intentions to change diet and exercise habits. This effect becomes more pronounced in women with higher appearance-based self-worth (McComb and Mills, 2022). In contrast, content focusing solely on fitness performance has been observed not to create such negative effects (Yan et al., 2025). Ahadi et al. (2025) have also revealed that women prioritizing a functional view of their bodies enables them to develop positive body perception even in competitive environments.

### 2.2 The role of personal trainers

The language and behaviors of personal trainers significantly affect women’s body image. Trainers’ respect for individual differences and focus on functionality contribute to women’s development of positive body image (Gray et al., 2025a), while perceived body acceptance contributes to increased body satisfaction (Gray et al., 2025b). These findings highlight the critical importance of a supportive training environment, especially for beginner women (Rosaini et al., 2026).

Conversely, negative experiences with trainers can have detrimental effects. Research has shown that when trainers focus excessively on weight loss, appearance, or criticize women’s bodies, it reinforces body dissatisfaction and reduces motivation to continue exercising (Mendes et al., 2024). Similarly, women who receive unsolicited comments about their appearance or performance from trainers often feel demoralized, with some even considering leaving the gym altogether (Cowley and Schneider, 2025). This is particularly concerning for beginner women, as negative early experiences may lead to exercise avoidance and reinforce existing barriers to physical activity (Khairunissya et al., 2025).

The quality of the trainer-client relationship is therefore critical. Trainers who adopt a client-centered approach, actively listen to women’s goals and concerns, and provide positive reinforcement have been shown to enhance women’s exercise self-efficacy and intrinsic motivation (Rosaini et al., 2026). Furthermore, trainers who model body acceptance behaviors-such as avoiding negative self-talk about their own bodies and focusing discussion on functional outcomes rather than appearance-can serve as powerful role models for their clients (Gray et al., 2025a). Mendes et al. (2024) also found that positive affect and reduced appearance self-consciousness are key mediators in the relationship between exercise and positive body image, suggesting that trainers who foster enjoyment rather than appearance-focused goals may yield better psychological outcomes. Given that a single comment from a trainer can affect a woman’s relationship with exercise for years, as highlighted in the present study’s findings, trainer training programs should include components on body-positive communication and awareness of gender-specific barriers.

### 2.3 Group exercise and program design

A qualitative study examining the experiences of premenopausal women participating in group exercise programs showed that women’s lived experiences, such as desire for exercise consistency and historically negative exercise relationships, shape the intervention process (Hadfield et al., 2025). Exercises that women find fun and that provide social connection and comfort were found to positively affect their exercise perceptions and strengthen their intentions to remain physically active (Golaszewski et al., 2023; Hadfield et al., 2025). Similarly, a study examining barriers to home-based physical activity showed that lack of motivation and social barriers are the strongest predictors of women’s physical activity levels (Noori-Sistani et al., 2025). These findings emphasize that women’s exercise programs should be designed considering their lived experiences and unique preferences (Wallbank et al., 2022).

### 2.4 The double bind of appearance-based exercise

When examining women’s attitudes toward appearance-based exercise, women are found to evaluate other women who engage in appearance-based exercise more critically than men do (Grahn, 2025). This reveals that women face a situation that can be called a “double bind”: they try to conform to both feminine and fitness norms, yet condemn other women who perform the same actions. This situation indicates that women’s physical appearance is a sensitive resource, constantly negotiated under the pressure of simultaneous social expectations. Supporting this finding, Cowley and Schneider (2025) demonstrated that women in gym settings frequently experience their bodies as “never enough” and feel “always on display,” navigating a tension between empowerment and objectification. Similarly, Limniou et al. (2025) found that exposure to fitspiration content on social media platforms such as TikTok significantly decreases body satisfaction among female viewers, reinforcing the double bind women face in fitness contexts.

### 2.5 Empowerment through exercise

A recent phenomenological study examining the exercise experiences of physically fit women showed that women’s narratives extend from persisting in exercise despite social expectations and sexist stereotypes to the desire for a long, happy, and healthy life (Deininger and Teriba, 2025). The study identified eight main themes: initial motivations for exercise, benefits of exercise, persistence, definition of a successful workout, gender-based barriers, physical fitness identity, cost of fitness pursuit, and appearance motives. The findings reveal how physical exercise promotes empowerment and psychological well-being in women, with benefits such as self-confidence and self-love supporting sustainable motivation. This aligns with the work of Amato et al. (2025), who reported that over 90% of female CrossFit® participants perceived improvements in self-esteem and daily energy, with 95% reporting reduced stress levels, suggesting that structured exercise programs can serve as powerful vehicles for psychological empowerment.

Furthermore, another qualitative study with women participating in strength training indicated that health problems and aging are the main sources of motivation, while the biggest barriers to participation are time constraints, lack of motivation, and cultural stigma (Rosaini et al., 2026). Extending these findings, Vasudevan and Ford (2022) identified that women’s initiation and maintenance of strength training is additionally influenced by lack of knowledge, inadequate support, caring responsibilities, and limited access to facilities, while key enablers include social support, observable mental and physical health improvements, and proper supervision. A’aqoulah et al. (2026) further highlighted that expensive gym memberships and a lack of women-only clubs represent significant structural barriers, though women consistently report exercising to boost self-satisfaction and self-confidence. Collectively, these studies demonstrate that while appearance-based pressures, cultural stigmas, and structural barriers persist as significant obstacles, exercise simultaneously serves as a powerful pathway to empowerment, self-acceptance, and long-term well-being for women.

A study examining the experiences of female Olympic weightlifters as they balance power, competitiveness, and esthetics when choosing weight classes showed that women frequently refer to sport-specific and gender-based body discourses, but that the body itself is also a determining factor (Nelson and Jette, 2023). The authors suggest that awareness of the “rebellious body” should be considered an important element of female athletes’ bodily empowerment. This finding demonstrates the complexity of women’s relationship with their bodies and the potential for the body to transform from a passive object into an active subject. Extending this perspective, research on women in strength sports more broadly has shown that engaging with physical strength can serve as a vehicle for developing body satisfaction, personal agency, and transformative empowerment (Gravelle et al., 2025; Shaheen, 2025). Together, these studies highlight that when women engage in strength-based physical activities, the body can shift from being an object of external surveillance to a source of subjective power and self-definition.

### 2.6 The present study

While all these studies reveal the complex and multidimensional nature of women’s fitness experiences, studies focusing on the unique experiences of women new to fitness are limited (Deininger and Teriba, 2025; Rosaini et al., 2026). Yet the challenges faced by women taking their first steps into the gym environment, the transformations they experience, and the coping strategies they develop warrant a separate investigation, both theoretically and practically. Existing research generally covers already active women (Nelson and Jette, 2023; Deininger and Teriba, 2025) or focuses on the effects of digital content (Grahn, 2025; Yan et al., 2025), failing to holistically address the “first step” experience of beginners (Csontos et al., 2026; Rosaini et al., 2026). Aiming to fill this gap in the literature, this study examines the gym experiences of women newly starting fitness from a phenomenological perspective. Accordingly, the research seeks answers to six fundamental questions: (1) What motivations drive women newly starting fitness to take action? (2) How do their body perceptions change during this process? (3) What concrete contributions does fitness make to their daily lives? (4) How do their aging perceptions transform? (5) How do the gym environment and social circle shape this experience? (6) What types of changes do all these experiences cause in their self-narratives? The study was conducted in Malatya, Türkiye, a mid-sized city where traditional gender norms regarding women’s roles and public presence remain influential. In this context, women’s participation in physical activity-particularly in gym settings-can be constrained by cultural expectations related to modesty, family approval, and the limited availability of women-only sports facilities. Women-only gyms thus serve not only as exercise spaces but also as rare public arenas where women can engage in physical activity without male scrutiny. This cultural backdrop is essential for interpreting participants’ experiences, as the sense of safety and solidarity they described may be partly attributable to the women-only nature of the gyms in a context where co-ed environments might carry additional social risks.

## 3 Materials and methods

### 3.1 Research model

This study is a qualitative research designed within the framework of a phenomenological approach aimed at understanding the essence of human experience (Smith et al., 2022; Zahavi, 2025). Phenomenology aims to explore in depth the lived experiences of individuals regarding a particular phenomenon and to reveal the common meaning structures of these experiences (Van Manen, 2023). This design is the most appropriate method for exploring the subjective meanings of gym experiences of women new to fitness, their relationship with their bodies, and the transformation they undergo in this process.

One of the fundamental principles of phenomenological research is to understand participants’ experiences from their own perspectives and to avoid interfering with the natural flow of data by bracketing the researcher’s biases (Creswell and Clark, 2017). In this study, the researcher’s assumptions about her own fitness experiences were attempted to be bracketed by keeping a reflective journal before the data collection and analysis process.

### 3.2 Study group

Criterion sampling and maximum variation sampling, which are purposive sampling methods, were used together in the selection of participants (Patton, 2014). The common criteria determined within the scope of criterion sampling are as follows: (a) being female, (b) being 18 years of age or older, (c) having performed regular fitness training for at least three days a week for at least two months, and (d) volunteering to participate in the study. Among the participants meeting these criteria, it was aimed to include women with different ages, education levels, occupational groups, fitness experience durations, and members of different fitness centers through maximum variation sampling.

The research was conducted in multiple women-only fitness centers operating in the province of Malatya, Türkiye. The ages of the participants ranged from 23 to 48, with a mean age of 28.1 (SD = 6.3). The most distinctive feature of the sample is that the vast majority of the participants (75%, *n* = 12) have been doing fitness for 12 months or less. The mean experience duration was calculated as 16.2 months (SD = 21.3), but the median value is 5.5 months. This indicates that the study group predominantly consists of “beginner” or “early stage” participants.

The principle of data saturation was taken as a basis in determining the sample size in qualitative research (Guest et al., 2020; Hennink and Kaiser, 2022). Written responses were terminated at the 16th participant, when no new themes emerged and data saturation was reached. This number is within the recommended sample size range for phenomenological research (Creswell and Poth, 2016; Smith et al., 2022). Detailed information on the demographic and fitness characteristics of the participants is presented in Table 1.

**Table 1:** Demographic and fitness characteristics of participants.

Code	Interview date	Age	Marital status	Education	Fitness experience duration	Weekly frequency	Training type*
K1	6.04.2026	33	Married	High School	4 months	6 days	S, C
K2	6.04.2026	25	Married	Univ.	1 year	4 days	S, C
K3	7.04.2026	27	Married	High School	4 months	6 days	S, C
K4	7.04.2026	31	Married	Primary School	2 months	6 days	S, C
K5	8.04.2026	27	Single	Univ.	2 months	3 days	S, C
K6	8.04.2026	38	Single	Univ.	3 months	5 days	S, G, C
K7	9.04.2026	48	Married	Univ.	5 months	6 days	S
K8	9.04.2026	25	Single	Associate Degree	4 years	5 days	S, G, C
K9	10.04.2026	23	Single	Univ.	4 months	6 days	S, C
K10	11.04.2026	23	Single	High School	3 months	6 days	S, G
K11	11.04.2026	29	Married	Associate Degree	2 years	4 days	S, G, C
K12	12.04.2026	26	Single	Univ.	7 years	3–4 days	G, C
K13	12.04.2026	29	Single	Univ.	6 months	4 days	S
K14	13.04.2026	23	Single	Associate Degree	3 years	4 days	S
K15	13.04.2026	24	Single	Univ.	3 months	6 days	S, C, F
K16	15.04.2026	33	Single	Univ.	6 months	4 days	S, G, C

*S, Strength; G, Group Classes; C, Cardio; F, Functional; Univ., University.

### 3.3 Data collection tools and process

In this study, data were collected in two stages. Written forms were used in the first stage to determine the demographic characteristics of the participants, and in the second stage to explore their fitness experiences in depth.

#### 3.3.1 Demographic information form

A short form was used to determine the participants’ age, education, marital status, occupation, and fitness history (experience duration, weekly frequency, training type).

#### 3.3.2 Written interview form

The form, which constitutes the main data source of the research, consists of six open-ended questions developed in line with the principles of the phenomenological approach (Kallio et al., 2016). The questions were prepared to explore (1) the reasons for starting their fitness journey, (2) the change in their body perception during this process, (3) the concrete contributions of fitness to their daily lives, (4) the transformation in their perceptions of aging, (5) the effect of the gym environment and social environment on their experiences, and (6) the changes created by this entire process in their self-narratives. A pilot application was conducted with two participants to test the comprehensibility of the questions, and it was determined that no changes were needed.

#### 3.3.3 Data collection process

Data were collected between April 6–15, 2026. Participants did not allow audio recording; therefore, the interviews were conducted in writing, respecting their privacy. The participants were given the question forms and asked to write their answers in their own expressions. The decision to use written responses was driven by participants’ explicit privacy concerns; all participants declined audio recording but expressed willingness to share their experiences in writing. To maximize the descriptive richness of these written accounts, the open-ended questions were deliberately designed to be broad and reflective, inviting participants to elaborate on emotions, bodily sensations, and contextual factors. While written responses lack the spontaneity of spoken interviews, they allowed participants to articulate their experiences carefully and without the pressure of immediate verbal response-which may have enhanced depth for some participants. The written data were subsequently supplemented with member checking and peer debriefing to ensure interpretive fidelity. Participants completed the interview forms at the gym. The average response time was 35 min (range: 15–60 min).

### 3.4 Data analysis

The written responses given by the participants were transferred to word files on the computer to be made ready for analysis. In the analysis of the data, the six-phase thematic analysis method developed by Braun and Clarke (2021) was followed. First, to gain familiarity with the written responses, they were read several times by the researcher and initial impressions were noted. In the second phase, the data were divided into meaningful units and coded manually; with a data-driven approach, codes were derived directly from participant expressions. The coding process was carried out by taking printouts of the written responses, marking them with colored pencils, and adding margin notes. In the third phase, the generated codes were grouped according to their similarities and differences to determine potential themes and sub-themes; code lists and thematic maps were created manually. In the fourth phase, the determined themes were checked for consistency with the codes and the entire data set, and necessary corrections were made. In the fifth phase, the scope, boundaries, and essence of each theme were clarified, and explanatory names were given to the themes. In the final phase, the findings were structured within the framework of themes and sub-themes and written down. To increase the reliability of the analysis process, the codes and themes were discussed with a second researcher, and differences were negotiated until a consensus was reached. Throughout the analysis, an analysis log was kept in which all decisions and interpretations made were recorded.

To illustrate the coding process, we provide two concrete examples of how raw data were transformed into codes and subsequently grouped into themes. First, the participant statement ‘Now I can say ‘no’ when I need to, and I protect myself much better’ (K1, age 33) was coded as ‘boundary-setting,’ which was grouped under the sub-theme ‘Autonomy and Boundary Setting’ within the overarching theme ‘Transformation Materialized in Daily Life and Identity Construction.’ Second, the statement ‘I have become so much stronger. I can now easily perform movements that I could not even dream of doing before’ (K16, age 33) was coded as ‘physical competence,’ which was grouped under the sub-theme ‘Sense of Strength and Competence’ within the theme ‘Paradigm Shift in Body and Self-Perception.’ These examples demonstrate the analytical pathway from raw data to thematic interpretation and enhance the transparency of our analysis.

### 3.5 Trustworthiness of the research

To ensure validity and reliability in qualitative research, the four criteria suggested by Lincoln and Guba (1985) were taken as a basis. These criteria have been adapted in light of the current qualitative research literature (Nowell et al., 2017; Korstjens and Moser, 2018).

#### 3.5.1 Credibility

Various strategies were used to ensure that the findings reflect reality. Member checking was performed by sharing the summaries of the themes and comments resulting from the analysis with three participants, and their feedback was received (Birt et al., 2016). In addition, long-term interaction was established between the researcher and the participants to develop a trusting relationship, and peer debriefing was carried out by regularly discussing the analysis process with a researcher experienced in qualitative research.

#### 3.5.2 Transferability

To ensure the transferability of the findings to other contexts, thick description was provided. The demographic characteristics, fitness histories, and contextual factors of the participants were described in detail, and the findings were enriched with direct quotations (Younas et al., 2023). Furthermore, participants with different ages, education, occupations, and experience durations were included in the study using maximum variation sampling.

#### 3.5.3 Dependability

An audit trail was created to demonstrate the consistency of the research process. All stages of the research (raw data, coding notes, analysis decisions) were documented and stored. In addition, the researcher kept a reflexive journal, recording her assumptions, emotions, and decisions throughout the data collection and analysis process (Finlay, 2024).

#### 3.5.4 Confirmability

To demonstrate the objectivity of the findings, reflexive journaling and peer debriefing helped the researcher identify, acknowledge, and systematically reflect on her assumptions and potential influences throughout the research process, thereby enhancing the study’s confirmability. All analytical decisions were justified based on the data.

### 3.6 Ethical considerations

Before the research, necessary ethical approval was obtained from the Inönü University Social and Human Sciences Scientific Research and Publication Ethics Committee (2026, 5/50). The research was conducted in accordance with the ethical principles of the Declaration of Helsinki regarding research involving human participants.

All participants were given written and verbal information about the purpose, method, potential risks, and benefits of the research. It was explained that participation was entirely on a voluntary basis and that they could withdraw at any time without giving any reason. Following this information, written informed consent was obtained from all participants by signing an informed consent form.

To protect the confidentiality of participants’ identity, written response forms and their digital copies were stored on a password-protected computer accessible only to the researchers. In the reporting, pseudonyms K1, K2, K16 were used instead of the participants’ real names. Any information that could reveal the identity of the participants (special place names, names, etc.) was removed from the texts. Care was taken not to cause any physical or psychological harm to the participants during the research, and it was stated that the written response process could be terminated if discomfort was felt.

## 4 Results

### 4.1 Thematic analysis

The thematic analysis followed the six-phase procedure outlined by Braun and Clarke (2021). In the third phase, codes were grouped into potential themes based on shared meaning and conceptual coherence. For example, codes related to self-trust, mirror acceptance, and capability were united under the overarching theme of “Paradigm Shift in Body and Self-Perception” because they all reflected a movement from external (appearance-based) to internal (functionality-based) evaluative criteria. This processual logic guided the organization of all four themes. When Figure 1 is examined, it is seen that the research findings are structured under four main themes: (1) Driving Forces for Initiation: Health, Body, and Emotional Triggers, (2) Paradigm Shift in Body and Self-Perception, (3) The Dual Face of the Social Environment: Supportive Sanctuary and Normative Questioning, and (4) Transformation Materialized in Daily Life and Identity Construction. A total of 15 subcategories and 25 codes were formed under these themes. The thematic structure reveals a holistic transformation process, starting from the participants’ motivations for beginning fitness, moving through the transformation in their body perceptions, their experiences with the social environment, and ultimately the empowerment processes occurring in their daily lives and female identity.

**Figure 1:**
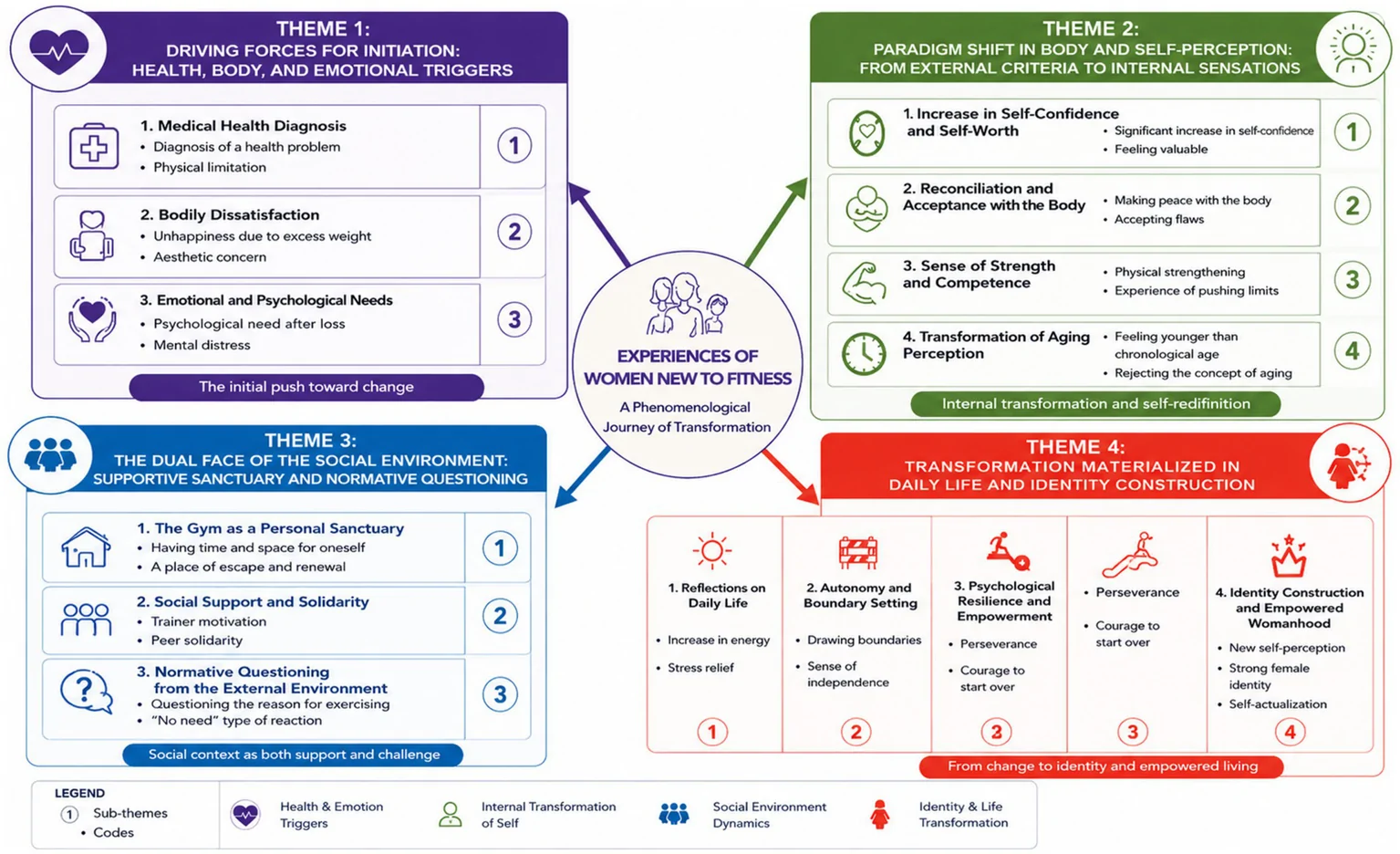
Thematic map of the experiences of women new to fitness. Arrows indicate the interaction between themes.

#### 4.1.1 Theme 1: driving forces for initiation: health, body, and emotional triggers

*Related interview question*: “How did this fitness journey of yours begin? What was it that first moved you to take action?”

This theme explains in depth the fundamental motivations that enabled the participants to begin their fitness journey. When the participants’ statements are examined, it is seen that their reasons for starting are shaped around three main categories: health-related concerns, bodily dissatisfaction, and emotional needs. Each of these three categories emerged in different ways within the participants’ own life experiences. Example participant quotes related to the findings are presented in Table 2.

**Table 2:** Driving triggers for initiation.

Category	Code	Example participant quote
Medical Health Diagnosis	Diagnosis of a health problem	*“When the internist told me that I had insulin resistance and that I needed to make lifestyle changes immediately, that’s when I decided to start exercising.” (K1, age 33)*
	Physical limitation	*“I had a meniscus tear and I could not even walk properly, let alone think about exercising. That physical limitation was the turning point for me.” (K7, age 48)*
Bodily Dissatisfaction	Unhappiness due to excess weight	*“I was extremely overweight and I was no longer happy with my body. I could not look at myself in the mirror without feeling uncomfortable.” (K4, age 31)*
	Aesthetic concern	*“After giving birth, I gained a lot of weight. I did not feel good about myself when I looked in the mirror, and that bothered me deeply.” (K3, age 27)*
Emotional/Psychological Need	Psychological need after loss	*“Losing my father was the most devastating event in my life. I needed to clear my mind, to escape from the constant sadness, and that’s why I started exercising.” (K6, age 38)*
	Mental distress	*“I was feeling constantly restless and uneasy for no particular reason. I needed something to calm my mind and regain my inner peace.” (K15, age 24)*

*Medical health diagnosis*: A significant portion of the participants stated that they decided to start fitness after receiving a diagnosis of a health problem or experiencing a physical limitation. For example, K1, aged 33, expressed that she decided to exercise after her internist diagnosed her with insulin resistance and told her that she needed to make lifestyle changes immediately. Similarly, K7, aged 48, stated that she could not even walk properly due to a meniscus tear, and that this physical limitation was the most important factor that moved her to take action. This situation shows that, for the participants, fitness initially functioned more as a treatment supplement or a health crisis preventer rather than a lifestyle choice. The participants perceived the doctor’s advice or the physical discomfort they experienced as a “wake-up call” and turned this external stimulus into an opportunity to take action.

*Bodily dissatisfaction*: The second important trigger was the discomfort participants felt with their own bodies. This dissatisfaction manifested itself across a wide spectrum, ranging from unhappiness caused by being overweight to a body image where one felt excessively thin. K4, aged 31, clearly stated that she was unhappy due to her excess weight and that this unhappiness was the main reason that led her to sports. She expressed that she could not look at herself in the mirror without feeling uncomfortable. On the other hand, K13, aged 29, experienced the opposite situation and stated that she started fitness because she was too thin. These statements from the participants suggest that the discrepancy between the idealized body image individuals have internalized and their perceived current body may create a strong psychological impetus to start fitness. This finding demonstrates the influence of societal beauty norms on women and shows that the effort to conform to these norms can be a source of motivation.

*Emotional and psychological needs*: Finally, it was observed that for some participants, the underlying reason for starting fitness was emotional and psychological needs. K6, aged 38, expressed that she started exercising to dispel the deep sorrow and confusion she experienced after her father’s death. Losing her father was the most devastating event in her life, and for her, fitness became an area where she could clear her mind and cope with her pain. K15, aged 24, stated that she turned to sports due to a more general feeling of restlessness, needing something to calm her mind and regain her inner peace. For these participants, fitness functioned as a coping mechanism and an emotional escape point, far beyond just being a physical activity. This finding suggests that the potential mental health benefits of physical activity may be experientially recognized by participants and transformed into an initial motivation, and can even be used as a form of “spontaneous therapy” in some cases.

#### 4.1.2 Theme 2: paradigm shift in body and self-perception: from external criteria to internal sensations

*Related interview questions*: “How has your view of yourself and your body changed since you started?” and “How old do you feel? How has fitness changed the concept of ‘aging’ for you?”

This theme explains in depth the profound transformation that the fitness experience has created in the participants’ body and self-perceptions. The most striking finding is the fundamental change in how the participants evaluate themselves and their bodies. Over time, it is observed that the criteria for evaluation shift from external, esthetically oriented concerns to internal, functional, and emotional sensations. The participants have begun to focus on “how they feel” and “what their bodies can do” rather than “how they look.” Example participant quotes related to the findings are presented in Table 3.

**Table 3:** Transformation in body and self-perception.

Category	Code	Example Participant Quote
Increase in Self-Confidence and Self-Worth	Significant increase in self-confidence	*“First of all, my self-confidence increased. Now I trust myself much more and I’m not afraid to try new things because I know I am capable of handling challenges.”* (K1, age 33)
	Feeling valuable	*“My self-confidence rose as if I were the only beautiful woman in the world. When I look in the mirror now, I actually like what I see, and that is a wonderful feeling that I had not experienced in years.”* (K4, age 31)
Reconciliation and Acceptance with the Body	Making peace with the body	*“I can say that I have made peace with my body. Day by day, I have become more reconciled with mirrors. Now I accept my body as it is, without constantly criticizing every little imperfection.”* (K9, age 23)
	Accepting flaws	*“I learned not to punish my body anymore. I learned to love it with all its shapes and flaws. I realized that I do not have to be perfect to be worthy or happy.”* (K9, age 23)
Sense of Strength and Competence	Physical strengthening	*“I have become so much stronger. I can now easily perform movements that I could not even dream of doing before, and that gives me an incredible sense of accomplishment.”* (K16, age 33)
	Experience of pushing limits	*“I have seen that as you push your limits, new strengths within you are revealed. I discovered how truly strong my body is, far beyond what I had ever imagined.”* (K16, age 33)
Transformation of Aging Perception	Feeling younger than chronological age	*“Before sports, my body felt 10 years older than it actually was. Now I feel much more energetic and youthful than my peers, almost like I’ve turned back the clock.”* (K1, age 33)
	Rejecting the concept of aging	*“The concept of aging loses its meaning with this sport. I do not think about my age anymore; I only think about my energy, my vitality, and what my body can still achieve.”* (K6, age 38)

*Increase in self-confidence and self-worth*: Almost all participants stated that there was a significant and positive increase in their self-confidence after starting fitness. This increase was not limited to the gym environment but also reflected in other areas of their lives. K1, aged 33, expressed that the lack of self-confidence she felt at the beginning of the process was replaced over time by a strong sense of self-trust, saying, “First of all, my self-confidence increased. Now I trust myself much more and I’m not afraid to try new things because I know I am capable of handling challenges.” K4, aged 31, described this change in more dramatic terms, stating, “My self-confidence rose as if I were the only beautiful woman in the world. When I look in the mirror now, I actually like what I see, and that is a wonderful feeling that I hadn’t experienced in years.” These statements suggest that the experience of setting a goal, striving to achieve it, and pushing physical limits may nourish the individual’s self-efficacy belief, which naturally and positively reflects on their overall level of self-confidence.

*Reconciliation and acceptance with the body*: Another significant transformation experienced by the participants was the qualitative change in their relationship with their bodies. While many participants had a problematic relationship with their bodies before fitness, it was observed that with regular exercise, this relationship was replaced by acceptance and even love. K9, aged 23, described this process very strikingly: “I can say that I have made peace with my body. Day by day, I have become more reconciled with mirrors. Now I accept my body as it is, without constantly criticizing every little imperfection.” She also added, “I learned not to punish my body anymore. I learned to love it with all its shapes and flaws. I realized that I don’t have to be perfect to be worthy or happy.” These statements reflect the healthy developmental stages in the body image literature and represent the transition of the body from a passive object or an criticized “enemy” to an “ally” with whom one dialogues, seeks to understand, and trusts.

*Sense of strength and competence*: Another dimension of the transformation, particularly prominent among participants who performed strength-based exercises such as weight training, was a distinct sense of strength and competence. K16, aged 33, expressed this experience very clearly: “I have become so much stronger. I can now easily perform movements that I couldn’t even dream of doing before, and that gives me an incredible sense of accomplishment.” He also said, “I have seen that as you push your limits, new strengths within you are revealed. I discovered how truly strong my body is, far beyond what I had ever imagined.” Here, it is observed that the value judgment is based on what the body can do, the concept of “capability,” rather than on how the body looks. This situation emerges as an extremely empowering experience, especially for women living in an appearance-oriented society.

*Transformation of aging perception*: Another striking outcome of the fitness experience was the shift in participants’ perceptions of aging and their subjective (felt) age. Many participants stated that they felt younger than their chronological age. K1, aged 33, said, “Before sports, my body felt 10 years older than it actually was. Now I feel much more energetic and youthful than my peers, almost like I’ve turned back the clock,” indicating that before fitness, her body made her feel 10 years older, but with regular exercise, her body now feels the same age as her spirit. K7, aged 48, stated that while she used to feel like she was 58, she now feels her actual age of 48 thanks to training. K9, aged 23, reinforced this by saying she feels like she is 18 rather than her chronological age of 23. Some participants questioned the concept of aging on a more fundamental level. K6, aged 38, argued that the concept of aging completely loses its meaning with fitness, stating, “The concept of aging loses its meaning with this sport. I don’t think about my age anymore; I only think about my energy, my vitality, and what my body can still achieve.” Meanwhile, K11, aged 29, went even further, using the powerful expression, “Aging is a lie that people have been made to believe.” Such radical statements suggest that participants perceived aging as a process that could be influenced through active participation, regular bodily care, and mental attitude, rather than viewing it as an inevitable and passive biological destiny. However, these perceptions reflect the subjective experiences of this specific sample and should not be generalized as universal outcomes of fitness participation.

#### 4.1.3 Theme 3: the dual face of the social environment: supportive sanctuary and normative questioning

*Related interview question*: “How do you feel in the environment where you exercise? What kinds of reactions do you get from those around you?”

This theme illustrates that the participants’ fitness experience was not independent of the social context and that they described experiencing a distinct dilemma between a safe internal environment and a questioning external environment. The women-only gym serves, on one hand, as a personal sanctuary and an area of empowerment for the participants. On the other hand, the questioning and criticisms based on traditional gender roles coming from the external social environment emerge as a significant factor shaping the participants’ motivation and experience. Example participant quotes related to the findings are presented in Table 4.

**Table 4:** Social environment experiences.

Category	Code	Example participant quote
The Gym as a Personal Sanctuary	Having time and space for oneself	*“I see the time I set aside for myself at the gym as the value I give to myself. This place is just for me, where nobody judges me, and I can finally breathe and focus on my own needs without any distractions.”* (K1, age 33)
	A place of escape and renewal	*“This is my escape and renewal place. It is an area where I get away from the stress of the day, where no one interrupts me, and I can focus solely on myself, my body, and my thoughts.”*(K14, age 23)
Social Support and Solidarity	Trainer motivation	*“My trainers motivated me so much. Sometimes when I felt like I was about to give up, their encouragement, guidance, and belief in me helped me continue and push through the tough moments.”* (K6, age 38)
	Peer solidarity	*“Everyone here is like friends, like siblings to each other. We sweat together, we struggle together, and we inspire each other. Knowing that I am not alone in this journey makes all the difference.”* (K9, age 23)
Normative Questioning from the External Environment	Questioning the reason for exercising	*“I frequently encounter questions from my close circle, including my husband, asking, ‘Why do you go to the gym even though you are thin?’ As if the only reason a woman could ever exercise is to lose weight, and nothing else matters.”* (K1, age 33)
	“No need” type of reaction	*“I receive reactions like, ‘You do not need to exercise, why are you doing it?’ Sometimes these comments genuinely upset me because they completely dismiss my personal reasons and motivations for wanting to be healthy and strong.”* (K5, age 27)

*The gym as a personal sanctuary*: The women-only gym was perceived by participants as a safe space where they felt free from judgment, performance anxiety, and the potential feeling of being scrutinized—a perception grounded in participants’ accounts rather than a direct comparison with co-ed environments. The participants described this space not merely as a place to exercise, but as a special area where they can allocate time for themselves, escape from the stress of daily life, and focus on their personal care. K1, aged 33, described this space as, “I see the time I set aside for myself at the gym as the value I give to myself. This place is just for me, where nobody judges me, and I can finally breathe and focus on my own needs without any distractions.” K14, aged 23, characterized it as, “This is my escape and renewal place. It is an area where I get away from the stress of the day, where no one interrupts me, and I can focus solely on myself, my body, and my thoughts.” K8 expressed the strength of the sense of belonging she established at this gym by saying, “I feel like I am exactly where I am supposed to be.” These statements show that the gym transcends being merely a physical space, transforming into an emotional sphere where participants feel safe, accepted, and valuable (Table 4).

*Social support and solidarity*: This safe space has also become more meaningful through a strong network of social support and solidarity. Participants emphasized the critical role of support they received from both trainers and other female participants in maintaining their fitness experiences. K6, aged 38, drew attention to the encouraging and guiding role of trainers by stating, “My trainers motivated me so much. Sometimes when I felt like I was about to give up, their encouragement, guidance, and belief in me helped me continue and push through the tough moments.” K9, aged 23, described the positive atmosphere created by peer solidarity, saying, “Everyone here is like friends, like siblings to each other. We sweat together, we struggle together, and we inspire each other. Knowing that I am not alone in this journey makes all the difference.” This supportive environment not only facilitated the participants’ exercise but also provided them with motivation and courage not to give up during challenging times. The participants stated that being part of this environment relieved them from feeling alone and made their fitness journeys more enjoyable.

*Normative questioning from the external environment*: However, in stark contrast to this supportive internal environment, participants expressed that they are frequently subjected to normative questioning from their external social circles (family, spouse, friends). The most striking questioning concerned the reasons why women exercise. Participants stated that they often face the question, “Why do you exercise?” especially when their physical appearance is perceived as “ideal.” K1, aged 33, described this questioning she encounters from her close circle, including her husband, by saying, “I frequently encounter questions from my close circle, including my husband, asking, ‘Why do you go to the gym even though you are thin?’ As if the only reason a woman could ever exercise is to lose weight, and nothing else matters.” K5, aged 27, stated that she received a more direct reaction, saying, “I receive reactions like, “You don’t need to exercise, why are you doing it?’ Sometimes these comments genuinely upset me because they completely dismiss my personal reasons and motivations for wanting to be healthy and strong.” These statements of the participants reflect how society often perceives women’s sports-primarily as an aesthetic (weight loss, getting in shape) or medical (therapeutic) obligation-while reasons such as gaining strength, personal enjoyment, psychological development, or autonomy may be misunderstood or questioned because they fall outside this pattern. This suggests that, in this cultural context, women’s broader motivations for fitness are not always fully understood or accepted by their social circles.

While the overwhelmingly dominant narrative was one of empowerment and positive transformation, participants also reported ambivalent and challenging experiences. For instance, K5 (age 27) expressed emotional distress from external questioning: ‘I receive reactions like, “You don’t need to exercise, why are you doing it?” Sometimes these comments genuinely upset me because they completely dismiss my personal reasons for wanting to be healthy and strong.’ Similarly, several participants described initial anxieties about entering the gym environment, fears of not knowing how to use equipment, and concerns about being judged by other gym members despite the women-only setting. These accounts suggest that while the fitness journey was largely transformative, it was not without its tensions, doubts, and persistent social pressures.

#### 4.1.4 Theme 4: transformation materialized in daily life and identity construction

*Related interview questions*: “What is the most visible contribution of fitness to your daily life?” and “What kind of woman have all these experiences made you? What has been your biggest personal discovery?”

This theme shows how the effects of the fitness experience transcend the boundaries of the gym, materializing in the participants’ daily life practices and ultimately settling at the center of their identity narratives. For participants, fitness ceased to be merely a physical activity; over time, it became a life practice that they described as profoundly transforming their daily routines, energy levels, emotional states, understanding of discipline, and most importantly, their perceptions of themselves. Example participant quotes related to the findings are presented in Table 5.

**Table 5:** Transformation materialized in daily life and identity construction.

Category	Code	Example participant quote
Reflections on Daily Life	Increase in energy	*“My energy has increased tremendously. Before, I could not do anything during the day because I was so exhausted all the time, but now I can keep up with everything, from work to household chores, without feeling drained.”* (K2, age 25)
	Stress relief	*“Fitness has helped me relieve stress. After work, I used to feel so tense and anxious, but after exercising, I feel completely relaxed, as if I have let go of all the weight on my shoulders.”* (K2, age 25)
Autonomy and Boundary Setting	Drawing boundaries	*“I have become a woman who draws her own boundaries. Now I can say ‘no’ when I need to, and I protect myself much better than I ever did before, without feeling guilty about it.”* (K1, age 33)
	Sense of independence	*“I have become a more independent person. I make my own decisions now, and I do not have to answer to anyone for the choices I make about my own life and my own body.”* (K8, age 25)
Psychological Resilience and Empowerment	Perseverance	*“I have developed the ability to never give up, even during the toughest workouts. I have carried this determination and persistence into other areas of my life, whether it is work, relationships, or personal goals.”* (K8, age 25)
	Courage to start over	*“This has been a discovery that taught me not to give up, but to love starting over. I learned that even if I fall, I can get back up, dust myself off, and continue moving forward without shame or hesitation.”* (K5, age 27)
Identity Construction and Empowered Womanhood	New self-perception	*“I have rediscovered myself. I realized how strong I actually am, beneath all the doubts and fears that used to hold me back. I am not the same person I was before.”* (K9, age 23)
	Strong female identity	*“I have become a much stronger, more self-confident woman. I know now that no difficulty or challenge can break me, because I have already overcome so much to get to where I am today.”* (K16, age 33)
	Self-actualization	*“Fitness has made me a peaceful, happy woman who is finally at peace with herself. Now I accept my body exactly as it is, without constantly criticizing every little flaw or wishing it were different.”* (K3, age 27)

*Reflections on daily life*: Participants reported tangible benefits after fitness, such as increased energy and reduced stress. K2, aged 25, described her transformation by saying, “My energy has increased tremendously. Before, I couldn’t do anything during the day because I was so exhausted all the time, but now I can keep up with everything, from work to household chores, without feeling drained.” K14, aged 23, stated that she feels “more energetic during the day and mentally calmer.” These findings show that fitness contributes not only to physical well-being but also to mental well-being.

*Autonomy and boundary setting*: Through fitness, participants developed the ability to set their own boundaries and have more say over their lives. K1, aged 33, expressed this transformation by saying, “I have become a woman who draws her own boundaries. Now I can say ‘no’ when I need to, and I protect myself much better than I ever did before, without feeling guilty about it.” K8, aged 25, reinforced this sense of independence by stating, “I have become a more independent person. I make my own decisions now, and I don’t have to answer to anyone for the choices I make about my own life and my own body.” K8 also emphasized the importance of taking time for herself by saying, “I see the time I set aside for myself as the value I give to myself.” These findings reveal that fitness strengthens individuals’ sense of autonomy.

*Psychological resilience and empowerment*: Participants stated that through fitness, their ability to cope with challenges improved and their psychological resilience increased. K8, aged 25, emphasized this psychological resilience by saying, “I have developed the ability to never give up, even during the toughest workouts. I have carried this determination and persistence into other areas of my life, whether it is work, relationships, or personal goals.” K5, aged 27, expressed that she developed a persistent attitude in the face of failure by stating, “This has been a discovery that taught me not to give up, but to love starting over. I learned that even if I fall, I can get back up, dust myself off, and continue moving forward without shame or hesitation.” K14, aged 23, highlighted the improvement in her stress management by saying, “I cope with stress much better now; I am calmer.” These findings show that fitness increases individuals’ psychological resilience.

*Identity construction and empowered womanhood*: The deepest outcome of all these experiences is the transformation participants experienced in their female identities. K9, aged 23, expressed that she has reached a new self-perception by saying, “I have rediscovered myself. I realized how strong I actually am, beneath all the doubts and fears that used to hold me back. I am not the same person I was before.” K16, aged 33, emphasized her strong female identity by stating, “I have become a much stronger, more self-confident woman. I know now that no difficulty or challenge can break me, because I have already overcome so much to get to where I am today.” K3, aged 27, expressed her sense of self-actualization and satisfaction by saying, “Fitness has made me a peaceful, happy woman who is finally at peace with herself. Now I accept my body exactly as it is, without constantly criticizing every little flaw or wishing it were different.” In conclusion, for participants in this study, fitness ceased to be a simple physical activity; it was experienced as a meaningful process of identity transformation and empowerment, through which they perceived themselves as stronger, more self-confident, more autonomous, and as more active agents of their own lives. These perceptions, however, reflect the subjective experiences of this sample and may not be generalizable to all beginner women.

## 5 Discussion

Before discussing the implications, it is important to note that the interpretations presented below are context-dependent and reflect the specific experiences of this sample. The findings offer rich phenomenological insight rather than universally generalizable truths. This study, by examining the experiences of women newly starting fitness through a phenomenological approach, has revealed that participants experienced this process as a multidimensional psychosocial transformation far beyond mere physical activity. The research findings show significant parallels with current studies aimed at understanding women’s experiences in gym settings, while also shedding light on the unique and in-depth experiences of women taking their first steps into their fitness journey. The diversity of participants’ motivations for starting fitness and how these motivations transform over time stands out among the findings. In this context, the discussion of the research is shaped around four main themes: initial motivations and health-related triggers, the paradigm shift in body and self-perception, the dual face of the social environment, and identity construction and empowerment.

### 5.1 Theme 1: initial motivations and health-related triggers

The initial motivations emerging in Theme 1 (medical diagnosis, bodily dissatisfaction, emotional needs) demonstrate the diversity of women’s reasons for starting sports. This finding aligns with Rosaini et al. (2026). In our study, emotional needs (coping after loss, general restlessness) were reported as just as strong a starting reason as health concerns. This finding suggests that, for this sample, women used fitness not only for physical health but also as a form of emotional coping-what some participants described as “spontaneous therapy.” However, the extent to which this applies to other populations requires further investigation. The moment of medical diagnosis served as a critical “trigger event” for sustainable behavior change, consistent with previous research (Csontos et al., 2026; Rosaini et al., 2026). Evaluated within the framework of objectification theory, the processes that began with external motivations such as medical diagnosis reflect our participants’ desire to have a healthy body rather than seeing their bodies as objects (Fredrickson and Roberts, 1997; Paulisova and Orosova, 2023).

### 5.2 Theme 2: paradigm shift in body and self-perception

The transformation in body perception demonstrates a paradigm shift from external (appearance) to internal (functionality, sensation) criteria. As beginner women focused on their own performance, they moved away from appearance concerns and reported developing a healthier relationship with their bodies. This finding is consistent with Yan et al. (2025), who found that performance-oriented fitness content does not negatively affect women’s body esteem. The process of reconciliation and acceptance with the body aligns with Gray et al. (2025b) and Ahadi et al. (2025), who revealed that prioritizing a functional view of the body enables positive body perception. Nelson and Jette's (2023) concept of the “rebellious body” provides a theoretical framework for understanding participants’ experiences of pushing their bodily limits. From the perspective of self-determination theory, the new relationship with their bodies indicates that their needs for competence, autonomy, and relatedness were being met (Deci and Ryan, 2002; Ntoumanis and Moller, 2025).

### 5.3 Theme 3: the dual face of the social environment

The social environment dilemma reveals the contradictory nature of women’s gym experiences. The women-only gym functioned as a “counter-space,” highlighting the importance of supportive environments (Cowley and Schneider, 2025). Social support from trainers and peers enhanced participants’ motivation, consistent with previous research (Golaszewski et al., 2023; Gray et al., 2025a; Hadfield et al., 2025). On the other hand, questions from the external environment reflect a tendency, at least within this cultural context, to frame women’s sports primarily in terms of aesthetic concerns, confirming the “double bind” experience of women (Grahn, 2025). It is important to note that our findings regarding the women-only gym as a “safe sanctuary” are derived from participants’ subjective accounts and should not be interpreted as evidence that co-ed gyms are inherently unsafe. Comparative research is needed to examine how different gym environments shape women’s experiences.

### 5.4 Theme 4: identity construction and empowerment

The identity construction and empowerment process emerging in Theme 4 constitutes an important contribution of our study. Beginner women in this sample reported developing autonomy, boundary-setting, and psychological resilience within a short period, suggesting the transformative potential of fitness within supportive environments. This aligns with Cowley and Schneider's (2025) findings that women experience empowerment through skill acquisition and breaking gender norms, and with Deininger and Teriba's (2025) work on psychological empowerment through exercise. Our participants’ statements about boundary-setting illustrate that they perceived themselves as having gained more control over their own lives and as being able to resist external expectations-a process that satisfies the need for autonomy central to self-determination theory (Deci and Ryan, 2002).

While the dominant narrative was one of empowerment, participants’ experiences were not uniformly positive. Several described persistent tensions: emotional distress from external questioning, initial anxieties about entering the gym environment, and the effort required to sustain motivation in the face of social norms that limit women’s exercise to aesthetic concerns. These ambivalent experiences align with Cowley and Schneider's (2025) observation that women in gym settings often navigate a tension between empowerment and objectification. Our findings suggest that empowerment through fitness coexists with doubts, social pressures, and moments of frustration. Acknowledging these complexities provides a more nuanced understanding of women’s fitness journeys and highlights the need for interventions that support not only physical progress but also emotional resilience.

### 5.5 Theoretical and practical implications

#### 5.5.1 Theoretical implications

This study makes several contributions to objectification theory and self-determination theory (Fredrickson and Roberts, 1997; Deci and Ryan, 2002).

First, it extends objectification theory by demonstrating how beginner women’s transition from appearance-based to function-based body perception unfolds in real time. The fitness environment-when supportive and women-only-can serve as a context for reducing self-objectification and cultivating body appreciation, aligning with Nelson and Jette's (2023) concept of the “rebellious body.”

Second, this study contributes to self-determination theory by identifying how satisfaction of basic psychological needs-particularly competence and autonomy-unfolds in the context of beginner women’s fitness journeys. Participants’ descriptions of gaining strength, setting boundaries, and receiving support provide rich qualitative evidence for how these needs are met in practice.

Third, this study bridges both theories by demonstrating that reduced self-objectification and increased need satisfaction are intertwined processes. When women shifted from appearance-focused to function-focused motivation, they simultaneously experienced greater autonomy and competence.

Fourth, this study contributes to the literature on women’s embodied experiences by documenting the transformation of aging perceptions among beginner women. Nishimura et al. (2026) found that long-term resistance trainers feel younger than their chronological age; our study extends this finding by showing that such shifts in subjective age can emerge within months of beginning fitness.

#### 5.5.2 Practical implications

The findings offer evidence-based strategies for fitness center managers, trainers, health professionals, and policymakers.

For fitness center managers and trainers, the finding that women-only gyms function as “safe sanctuaries” indicates that dedicated spaces or times for women, particularly beginners, should be considered. Where women-only facilities are not feasible, women-only hours, orientation sessions, or small group classes for beginners can be implemented. The paradigm shift from appearance-based to function-based body perception suggests that trainers should deliberately cultivate this shift through their language and feedback-asking questions such as “How did that feel?” rather than commenting on appearance, and providing feedback on performance improvements rather than physical changes. The importance of social support highlights the value of group-based programs, buddy systems, and online support groups for enhancing motivation and persistence (Golaszewski et al., 2023; Hadfield et al., 2025).

For health professionals and exercise program designers, the diversity of initial motivations-ranging from medical diagnoses to emotional needs to bodily dissatisfaction-indicates that exercise programs should not adopt a “one-size-fits-all” approach. Tailoring recommendations to individual motivations may be more effective. The finding that beginner women develop autonomy, boundary-setting skills, and psychological resilience within a short period suggests that exercise programs should explicitly incorporate elements that support these outcomes, such as goal-setting workshops, self-monitoring tools, and reflection exercises.

For policymakers and public health campaigns, the external questioning participants faced reflects persistent societal norms that limit women’s sports to aesthetic concerns. Public health campaigns should challenge these norms by emphasizing the multidimensional benefits of exercise for women: strength, mental health, autonomy, stress relief, and identity transformation. School curricula should help girls develop positive, functional relationships with their bodies from an early age, focusing on what the body can do, the joy of movement, and exercise as self-care.

### 5.6 Limitations and suggestions for future research

The findings should be evaluated within the context of several methodological limitations.

First, the sample consists predominantly of young adults (aged 23–33) from women-only gyms in Malatya, Türkiye, limiting transferability to other age groups, co-ed environments, and different cultural contexts. Future research should include more diverse samples across age, gender, and cultural settings.

Second, the cross-sectional design captures experiences at a single point in time; longitudinal research is needed to examine the permanence and long-term effects of the observed transformations.

Third, the study relied solely on self-reported written data; participants’ privacy concerns precluded audio recording. Future research could use multi-method approaches, including interviews, observational data, or daily diary methods. Additionally, data collection within the gym setting may have introduced social desirability bias.

Fourth, the study focused exclusively on women who had persisted in fitness for at least two months, excluding those who dropped out earlier. Consequently, our findings may reflect a predominantly positive picture of fitness experiences. Future research should include women who drop out early to capture the full spectrum of experiences, including barriers and negative encounters.

Future research could also explore the impact of digital platforms on beginner women’s experiences, examine the “rebellious body” concept through longitudinal or ethnographic methods, investigate the role of aging perceptions in women’s fitness motivation, conduct cross-cultural comparisons to identify universal versus culture-specific aspects of women’s fitness experiences, and extend these findings to other forms of physical activity beyond gym-based fitness.

## 6 Conclusion

This study examined the experiences of women new to fitness and revealed that participants experienced this process as a multidimensional psychosocial transformation. Women in this sample began their fitness journey due to medical diagnoses, bodily dissatisfaction, or emotional needs; over time, they reported increased self-confidence, making peace with their bodies, and a sense of strength and competence. Women-only gyms functioned as safe havens, while external questioning revealed the persistent influence of social norms limiting women’s sports to aesthetic concerns. Ultimately, participants perceived fitness as contributing to a stronger female identity by fostering autonomy, boundary-setting, and psychological resilience. These findings should be interpreted within the cultural and contextual boundaries of this study.

From a theoretical perspective, this study contributes to objectification theory and self-determination theory by documenting how beginner women’s transition from appearance-based to function-based body perception unfolds in real time. From a practical perspective, the results indicate that exercise programs should include holistic components that support psychosocial transformation, and trainers, gym managers, and policymakers should consider function-focused approaches and safe, supportive environments for beginners.

In conclusion, for women new to fitness, the journey is not merely about changing their bodies but about transforming their relationship with their bodies-from surveillance and criticism to dialogue, trust, and appreciation.

## Data Availability

The raw data supporting the conclusions of this article will be made available by the authors, without undue reservation.
